# Case report: Treatment-resistant depression, multiple trauma exposure and suicidality in an adolescent female with previously undiagnosed Autism Spectrum Disorder

**DOI:** 10.3389/fpsyt.2023.1151293

**Published:** 2023-04-26

**Authors:** Ilaria Secci, Lucie Petigas, Alexandra Cuenod, Paul Klauser, Carole Kapp, Audrey Novatti, Marco Armando

**Affiliations:** ^1^Section of Child and Adolescent Neuropsychiatry, Department of Public Health and Pediatric Sciences, University of Turin, Turin, Italy; ^2^Service of Child and Adolescent Psychiatry, Department of Psychiatry, Lausanne University Hospital and the University of Lausanne, Lausanne, Switzerland; ^3^Psychiatric Liaison Service, Department of Psychiatry, Lausanne University Hospital, Lausanne, Switzerland; ^4^Center for Psychiatric Neuroscience, Department of Psychiatry, Lausanne University Hospital and the University of Lausanne, Lausanne, Switzerland

**Keywords:** Autism Spectrum Disorder, depression, adolescent, case report, female, lithium, COVID-19, suicidality

## Abstract

High rates of co-occurring depression are commonly reported in youth with Autism Spectrum Disorder (ASD), especially in individuals without intellectual disability (ID). Depression in ASD undermines adaptive behavior and is associated with a higher risk of suicidality. Females with ASD may be particularly vulnerable due to their greater use of camouflaging strategies. Indeed, in comparison to males, ASD is underdiagnosed in females, despite higher rates of internalizing symptoms and suicidality. Trauma exposure may also play a role in the development of depressive symptoms in this population. Moreover, evidence for effective treatments of depression in autistic youth are lacking, with ASD individuals frequently experiencing low efficacy and side effects. We present the case of an adolescent female with previously undiagnosed ASD without ID, admitted for active suicidal plans and a treatment-resistant depression (TRD), occurred after a COVID-19 lockdown in the context of cumulative exposure to stressful life events. Comprehensive clinical assessments performed at intake confirmed severe depression with suicidality. Intensive psychotherapy and different changes in medications were carried out (SSRI, SNRI, SNRI + NaSSA, SNRI + aripiprazole), all of which were ineffective, with persistent suicidal thoughts, often requiring intensive individual monitoring. The patient was finally successfully treated with lithium augmentation of fluoxetine, with no side effects. During hospitalization she was also evaluated by an ASD specialized center, where a diagnosis of ASD was made according to the Autism Diagnostic Observation Schedule (ADOS) and the Autism Diagnostic Interview-Revised (ADI-R) scores, as well as to clinical judgment of a senior psychiatrist. The present case report shows that clinicians should not overlook undiagnosed autism as a possible cause of TRD, especially in females without ID, where higher rates of under diagnosis may be in part related to their greater use of camouflage. It also suggests that ASD underdiagnosis and resulting unmet needs may be involved in vulnerability to stressful experiences, depression, and suicidality. Furthermore, it shows the complexity of providing care to TRD in youth with autism, suggesting that an augmentation therapy with lithium, a commonly recommended therapeutic strategy for refractory depression in typically developing samples, may also be effective in this population.

## Introduction

Autism Spectrum Disorder (ASD) is a neurodevelopmental disorder characterized by deficits in social communication and interaction, and by restricted, repetitive patterns of behaviors and /or interests (1). High rates of co-occurring depression are commonly reported in youth with ASD (2, 3), with a lifetime prevalence rate estimated at 20.2% (4). ASD individuals are up to 4 times more likely to develop depressive symptoms than neurotypical subjects, with an increasing trend from adolescence to middle adulthood (5). Depression in ASD undermines adaptive behavior and is associated with a higher risk of suicidality and an increased healthcare burden (3).

A recent body of literature on adolescent and adult samples suggests that females with ASD may experience higher rates of depression and other internalizing symptoms compared to males, including anxiety, suicidality and eating disorders (5–7), although others studies found no sex differences (8, 9). Females with ASD also show higher rates of completed suicide than their male counterpart, in contrast to what is reported in non-ASD samples (i.e., completed suicide more frequent in males) (10). Moreover, evidence suggests that ASD is potentially underdiagnosed in females (11), who are also later diagnosed than their male counterpart and frequently misdiagnosed with other mental disorders, especially personality disorders (12–15). In line with this, some authors suggest that there's a female autism phenotype, including female specific manifestations of autism less likely to be captured by current diagnostic tools (16, 17). Also, females seem to mask more their autistic traits than males, a phenomenon known as camouflage (18), which has been associated to higher rates of distress, depression and suicidality in both adolescents and adults with ASD (19–21). Conversely, an earlier ASD diagnosis has shown a protective effect on depression and self-harm behaviors (22, 23), potentially enabling timely interventions and social support, and reducing the risk of traumatic experiences (24), that have been linked to mood symptoms and suicidality in ASD (25, 26).

Since current evidence is heavily based on male samples, providing information on the female autism phenotype could reduce mis- and missed-diagnosis rates and prevent secondary comorbidities in this population.

Moreover, evidence for efficacious pharmacological interventions is lacking and therapeutic strategies used for neurotypical patients may not be effective in ASD population (27, 28). Some authors suggest considering ASD in case of treatment-resistant depression (TRD) (29), which is defined by the presence of persistent depressive symptoms despite at least two trials of antidepressants at an appropriate dosage and duration. Lithium augmentation is a first-line treatment strategy for TRD in neurotypical samples (30). Moreover, lithium has demonstrated an anti-suicidal effect (31). Despite its potential use, to date, no randomized controlled trials have studied lithium's use in ASD, however, evidence from previous studies suggested a potential efficacy in this population. A preclinical study by Wu et al. (32) found an improvement in anxiety and depressive symptoms in rats with isolation-induced autistic behaviors. Previous chart reviews of youth and adults with ASD suggested a potential efficacy on mood dysregulation and maladaptive behaviors (33, 34). A previous case report showed a completed remission of catatonia and regression in two people with ASD with SHANK3 mutation treated with lithium (35). Epperson et al. (36) reported significant improvements in social relatedness and aggressivity with lithium augmentation of fluvoxamine in a man with ASD. Another case report documented a marked reduction in self-injury and aggressive behavior with lithium augmentation in a depressed adolescent male with intellectual disability (ID) (37). However, to our knowledge, this is the first case report of lithium augmentation efficacy in TRD with suicidality in an adolescent female with ASD, without ID. She was admitted multiple times for chronic suicidal plans and a TRD occurring after COVID-19 lockdown and exposure to traumatic events. Despite different changes in antidepressant treatments, all were ineffective. Eventually, she was diagnosed with ASD during her 4th hospital stay and successfully treated with lithium augmentation, without side effects.

## Case description

### Patient information

A. is a 16-year-old girl who was admitted four times in our adolescent psychiatric inpatient unit for depressive symptoms and active suicidal plans. The early development was normal, except for a mild language delay (i.e., she did not speak single words until 2.5 years and had poor speech until 4 years), that benefited from speech therapy. Since early childhood she showed difficulties in interacting with peers, isolation, peculiar interests (i.e., drawing letters and numbers), and was described by teachers as “being in her world,” which was interpreted as a reaction to the sudden death of her father when she was 5 years old. Moreover, she presented poor cognitive flexibility and a marked sense of justice, especially concerning school rules. Her peers frequently bullied her due to this. Also, the school context required her great effort due to her noise sensitivity. During adolescence, she reported a sense of being inadequate in social situations, so that she had to constantly monitor and adjust her reactions to fit into peer contexts. All her activities were organized with a rigid timetable, with a large amount of time dedicated to study and sport training, achieving brilliant sport and school results. Interactions with peers were mostly around these specific interests. Depressive symptoms started during the COVID-19 pandemic lockdown and the interruption of her high-performance sport training due to a sports failure. She presented low mood, fatigue, anhedonia, increased social withdrawal and ruminations about her father's death and the sports failure. She was first referred to an outpatient psychiatric unit, where she was treated with psychotherapy. Despite an initial mild improvement, she was later hospitalized four times for recurrent suicidal plans and diagnosed with major depressive disorder (MDD) according to DSM-5 criteria. During the first three admissions, several changes in medications were carried out: she was first treated with sertraline, up to 200 mg daily, that was then switched to fluoxetine, up to 20 mg daily, that was finally switched to duloxetine, up to 60 mg daily. She was also treated with mirtazapine up to 15 mg daily for her sleep disturbances, with a positive effect. The antidepressant treatments showed only a partial and temporary improvement, with a rapid recrudescence of suicidal plans after each hospitalization. A suspicion of ASD was also hypothesized during her first hospitalization, and the patient was put on a waiting list for outpatient specialized evaluations.

### Family history

There was no family history of psychiatric disorders, except a suspicion of autism in her small brother, who had never been investigated.

### Clinical findings

At the present hospitalization (i.e., the 4th one), a clinical interview was conducted at intake by a senior psychiatrist, confirming the diagnosis of MDD with active suicidal plans. She was also evaluated by the Prodromal Questionnaire-16 (38), with a score of 6 (cut-off 7) and by the Health of the Nation Outcome Scales for Children and Adolescents, self-rated version (HoNOSCA-SR) (39), suggesting severe depressive symptoms with suicidality (HoNOSCA-SR: “*Have you done anything to injure or harm your-self on purpose”- 4/5; “Have you been feeling in a low or anxious mood, or troubled by fears, obsessions or rituals”- 5/5*). During interviews, she reported depressed mood, guilt, anhedonia, apathy, sleep and appetite disturbances. She struggled to express emotions and her affect was blunted with limited eye contact, neutral facial expressions, and monotone speech. The intelligence quotient was not evaluated due to symptoms severity, however, she demonstrated good cognitive and linguistic competence, as evidenced by her school results and sophisticated vocabulary.

### Therapeutic intervention, follow-up, and outcomes

The current hospitalization was complicated by recurrent non-suicidal self-injuries (NSSI) (e.g., self-cutting) and suicidal threats, including an interrupted suicide attempt, requiring intensive individual monitoring during high-risk periods. A. also developed severe food restriction, initially describing it as her only way to die. She subsequently lost significant weight, reaching a body mass index of 16, and ultimately met all diagnostic criteria for anorexia nervosa (DSM-5), necessitating a nasogastric feeding tube.

**Figure 1:**
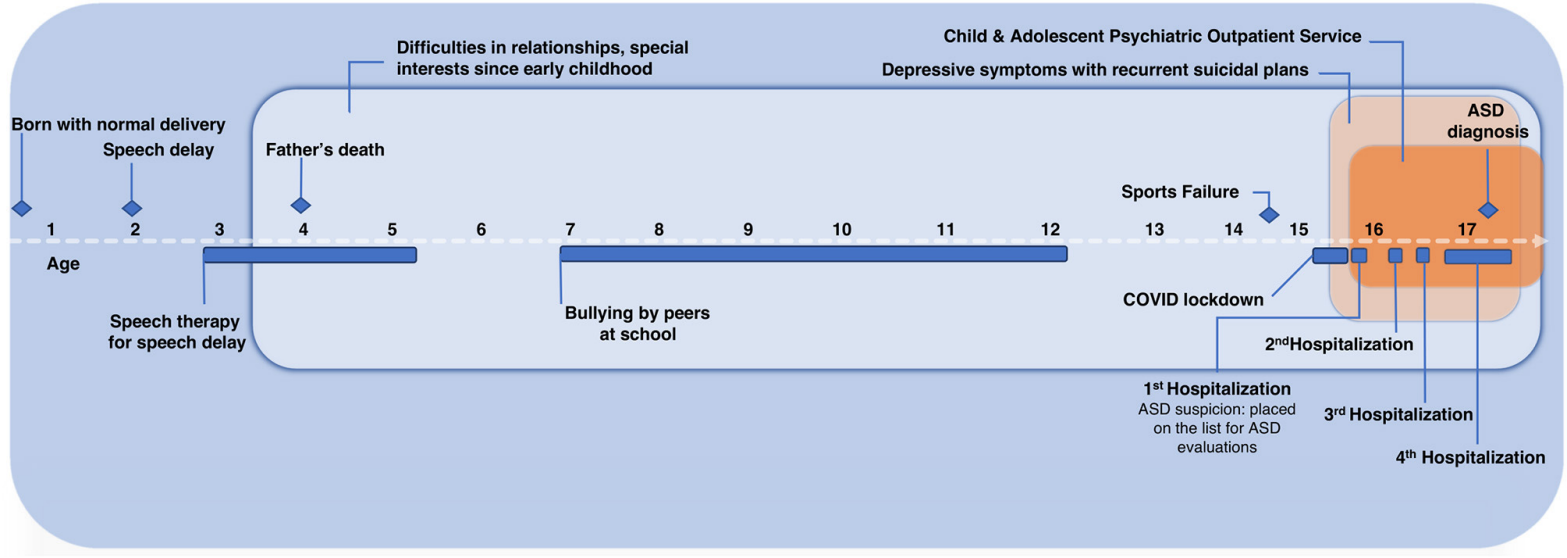
Case report timeline with relevant data.

**Figure 2:**
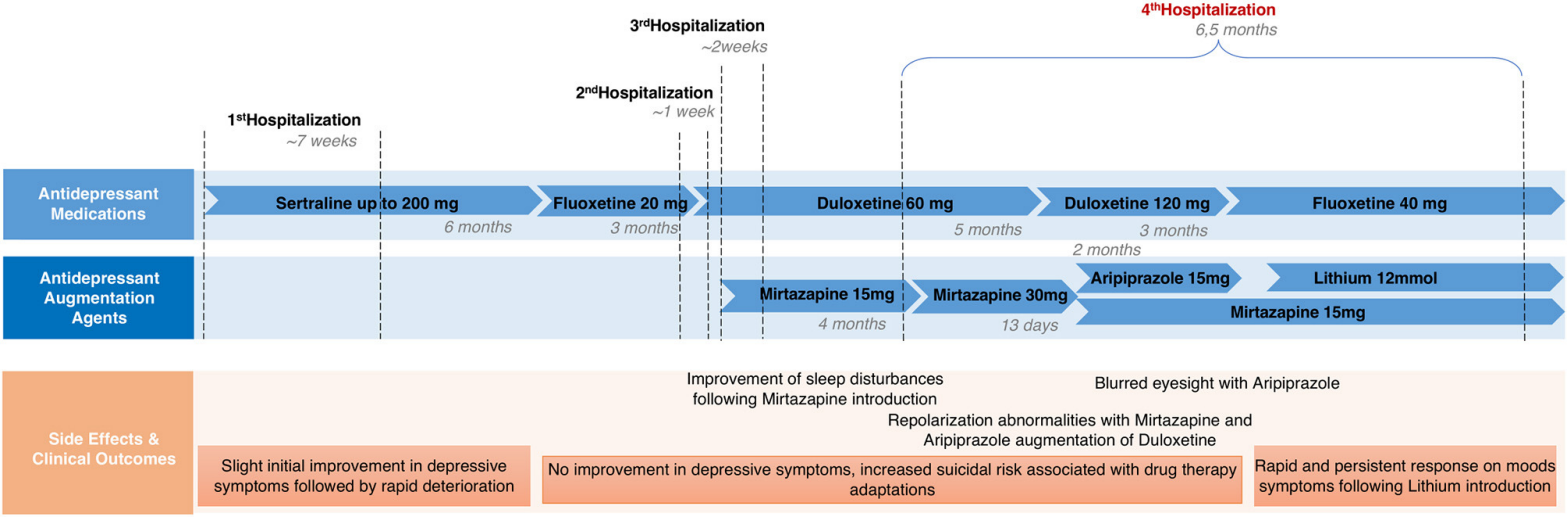
Patient's medication history.

Concerning pharmacological treatment, duloxetine was initially raised up to 120 mg daily, with no clinical response. Thus, an augmentation treatment, first with mirtazapine (up to 30 mg daily), and then with aripiprazole (up to 15 mg daily), was tried in combination with duloxetine, showing no clinical improvements. Duloxetine was then switched to fluoxetine, augmented up to 40 mg daily (since it had only been previously tried at a low dose), showing no clinical improvements. Lithium was finally added to fluoxetine as an augmentation treatment. Blood tests were conducted periodically to monitor thyroid, parathyroid, and renal function, as well as lithium plasma concentration, to determine the optimal dose and detect possible side effects. The therapeutic dose was 12 mmol daily (corresponding to a lithium plasma level of 0.5 mmol/L), split into two administrations, resulting in a rapid and satisfactory improvement in mood and suicidality, without side effects. A. also showed a parallel improvement in food intake restriction that allowed the removal of the nasogastric tube. During hospitalization, she was evaluated by an ASD specialized center (*Centre Cantonal de l'Autisme)* and diagnosed with ASD based on ADOS-2 and ADI-R scores (40, 41) (Table 1), as well as the clinical judgment of a senior psychiatrist. A brief psychoeducational intervention followed to help her elaborate the diagnosis. A. also had several ergotherapy sessions focused on recognizing and managing emotions. At hospital discharge, we observed a clinical improvement that was confirmed by the HoNOSCA-SR (with a score of 3/5 as regards to mood and anxiety symptoms, and a score of 3/5 as regards to suicidal thoughts). The patient was addressed to an outpatient clinic for psychological and psychiatric follow-up, as well as to a social skills training group for ASD. Clinically relevant data and medication history are shown in Figures 1, 2.

**Table 1:** Scores on the Autism Diagnostic Observation Schedule-2 (ADOS-2) and Autism Diagnostic Interview-Revised (ADI-R), with cut-off values.

**Scores and cut-offs on the ADOS-2 and ADI-R subscales**		
	**Score**	**Cut-off score**
**Autism Diagnostic Observation Schedule-2**		
Social affect	9	
Restrictive and repetitive behaviors	1	
Total	10	7
Comparison score	Moderate	
**Autism Diagnostic Interview-Revised**		
Reciprocal social interaction	12	10
Communication	9	8
Restrictive and repetitive behaviors	4	3
Development	5	1

## Discussion

We present the case of a TRD with NSSI, suicidal behaviors and anorexia nervosa in a 16-year-old-girl with comorbid previously undiagnosed ASD, without ID. The strength of our case report is that it highlights the clinical challenges of females with ASD, describing the factors possibly involved in both autism misdiagnosis and in the development of TRD with suicidality. To our knowledge, this is the first case report specifically exploring lithium augmentation's efficacy for TRD with suicidality in an adolescent female with ASD, without ID, suggesting a potential effectiveness. The limitation of our case report is that the evaluations for ASD were performed during hospitalization (albeit together with the improvement of depressive symptoms), therefore, the possible influence of depression on the scores may not be completely excluded. However, ASD suspicions preceded the current patient's hospitalization and the patient's history was thoroughly explored by clinicians with the aid of the ADI-R, a standardized tool providing a developmental perspective.

Our case is in line with the evidence suggesting a greater incidence of depressive symptoms, suicidality and eating disorders in ASD population, especially in females without ID (5, 42). Also, A. showed many typical characteristics of the female autism presentation, including a late ASD diagnosis, being initially referred for depression and using camouflage to fit in with peers (12, 16, 17). Additionally, she displayed “socially acceptable” repetitive interests (e.g., sport and school performance), consistent with studies indicating that females with ASD exhibit less bizarre interests and externalizing behaviors than their male counterpart, contributing to fewer mental health concerns and missed diagnosis (43, 44). Camouflaging strategies adopted by the patient, described as “constantly monitoring and adjusting her reactions to fit in social contexts,” may both have contributed to ASD misrecognition, and represented a risk factor for depression and suicidality, as evidence highlighted that camouflage is a risk factor for a wide range of mental health problems (16, 45). Despite being “exhausting and distressing,” qualitative studies in adolescent girls with ASD highlighted that camouflage was aimed at “fitting into” neurotypical contexts and avoiding bullying experiences (46). Traumatic experiences also seem to play a major role in the development of secondary comorbidities in ASD, including depression and suicidality (26, 47). Indeed, evidence show that individuals with ASD reported higher rates of trauma, such as bullying victimization and marginalization, compared to neurotypical peers, especially in females with ASD (48). Furthermore, typical autistic traits, including ruminative thinking, unusual sensory processing and need for predictability, may alter the appraisal of stressful events and hinder coping with changes. This may lead to a wider range of traumatic experiences and a greater impact on mental health (47, 49).

Multiple traumatic events in our patient's history, including sports failure and COVID-19 school closures, preceded the onset of depressive symptoms, and disrupted her routine, highlighting the impact of trauma and COVID-19 pandemic in depression development in ASD samples, as evidenced in previous studies and reports (26, 50–52). Also, COVID-19 lockdown may have exacerbated maladaptive coping strategies, such as ruminations on past traumatic experiences.

In sum, we hypothesize that the delayed diagnosis due to the female-typical ASD presentation, combined with exposure to multiple traumatic events, may have increased the risk of depression and suicidality in our patient. Furthermore, in line with literature findings (22), an earlier ASD diagnosis may have been a protective factor in reducing the patient's exposure to social stressors and in providing appropriate interventions and support. Indeed, evidence-based psychological treatments adapted for autism have shown efficacy for depression (53, 54).

Regarding pharmacological interventions, current evidence for effective treatments for depression in ASD is limited (27, 28). Studies exploring SSRI efficacy in reducing ASD core symptoms suggest that ASD youth may experience more adverse side effects than typically developing peers, especially behavioral activation (55, 56). In general population samples, current treatment guidelines recommend augmentation strategies after the failure of two antidepressants or a partial response with a primary antidepressant (57), with atypical antipsychotics and lithium commonly used as augmentation agents in both adults and adolescents (58, 59). Despite our patient's lack of response to various medications, including SSRI and SNRI monotherapy, as well as aripiprazole or mirtazapine augmentation, she showed improvement in depressive symptoms and suicidality with lithium augmentation. Limited evidence supports lithium's use in ASD, but previous clinical and pre-clinical studies documented its potential efficacy for mood symptoms, catatonia, social relatedness, and maladaptive behaviors in this population (32, 34, 36, 37). Our case also suggests lithium as a potential effective strategy for treating TRD and reducing suicide risk in youth with ASD.

## Conclusions

This case report has several clinical implications. First, it provides evidence on the female-typical ASD presentation, which could help recognize ASD in females and prevent secondary comorbidities. Second, it suggests that clinicians should not overlook undiagnosed autism as a possible cause of TRD with suicidality, especially in females without ID. Third, it suggests that an augmentation therapy with lithium, an agent commonly recommended for TRD in neurotypical samples, may also be considered for refractory depression in youth with ASD. Given the high rates of depression and suicidality in ASD and limited evidence for effective interventions, further research is needed to evaluate the efficacy of lithium augmentation strategy for TRD in this population. To address this gap in knowledge, prospective controlled trials with larger samples seem necessary.

## Patient perspective

The patient identified with the ASD diagnosis, describing difficulties in understanding other people's feelings or intentions since childhood, expressing relief when we discussed the increased prevalence of bullying among youth with autism. Also, she recognized the benefits of the augmentation therapy with lithium and was satisfied with the psychoeducational intervention that provided her with practical techniques to improve emotions recognition and expression.

## Data availability statement

The raw data supporting the conclusions of this article will be made available by the authors, without undue reservation.

## Ethics statement

Ethical review and approval was not required for the study on human participants in accordance with the local legislation and institutional requirements. Written informed consent to participate in this study was provided by the participants' legal guardian/next of kin. Written informed consent was obtained from the individual(s), and minor(s)' legal guardian/next of kin, for the publication of any potentially identifiable images or data included in this article.

## Author contributions

IS contributed to the conceptualization and writing of the manuscript. LP worked with the patient and her family and contributed to the writing. AC, LP, PK, and AN worked with the patient and her family, contributed to the conceptualization, and supervised the work. CK and MA contributed to the conceptualization and supervised the work. All authors have approved the submitted version.
